# Leaf and Life History Traits Predict Plant Growth in a Green Roof Ecosystem

**DOI:** 10.1371/journal.pone.0101395

**Published:** 2014-06-30

**Authors:** Jeremy Lundholm, Amy Heim, Stephanie Tran, Tyler Smith

**Affiliations:** 1 Departments of Biology and Environmental Science, Saint Mary’s University, Halifax, Nova Scotia, Canada; 2 Eastern Cereal and Oilseen Resarch Centre, Agriculture and Agri-Food Canada, Ottawa, Ontario, Canada; University of Oxford, United Kingdom

## Abstract

Green roof ecosystems are constructed to provide services such as stormwater retention and urban temperature reductions. Green roofs with shallow growing media represent stressful conditions for plant survival, thus plants that survive and grow are important for maximizing economic and ecological benefits. While field trials are essential for selecting appropriate green roof plants, we wanted to determine whether plant leaf traits could predict changes in abundance (growth) to provide a more general framework for plant selection. We quantified leaf traits and derived life-history traits (Grime’s C-S-R strategies) for 13 species used in a four-year green roof experiment involving five plant life forms. Changes in canopy density in monocultures and mixtures containing one to five life forms were determined and related to plant traits using multiple regression. We expected traits related to stress-tolerance would characterize the species that best grew in this relatively harsh setting. While all species survived to the end of the experiment, canopy species diversity in mixture treatments was usually much lower than originally planted. Most species grew slower in mixture compared to monoculture, suggesting that interspecific competition reduced canopy diversity. Species dominant in mixture treatments tended to be fast-growing ruderals and included both native and non-native species. Specific leaf area was a consistently strong predictor of final biomass and the change in abundance in both monoculture and mixture treatments. Some species in contrasting life-form groups showed compensatory dynamics, suggesting that life-form mixtures can maximize resilience of cover and biomass in the face of environmental fluctuations. This study confirms that plant traits can be used to predict growth performance in green roof ecosystems. While rapid canopy growth is desirable for green roofs, maintenance of species diversity may require engineering of conditions that favor less aggressive species.

## Introduction

Green roofs consist of vegetation and growing media, deployed on building rooftops. They are constructed to provide a range ecosystem services including reduction of stormwater runoff [1], reduction of energy fluxes through the building envelope [2], and provision of habitat for urban biodiversity [3], [4]. Green roofs with shallow growing media (usually less than 200 mm) are termed “extensive” green roofs, and are increasingly popular as they provide key services, while minimizing weight loading on buildings. Shallow growing media and exposed rooftop conditions are challenging for plant growth and survival, due to drought stress [5] and winterkill [6], thus the green roof industry relies on drought-tolerant plant species for these extensive green roofs.

Green roofs are commonly planted with succulents such as *Sedum* spp. However, there is a movement to incorporate other plant life-forms, such as grasses, to increase functional diversity [7], provide visual interest [5], and to provide better habitat for invertebrates [3]. Other life-form groups may not survive well, depending on roof conditions [8], [9]. Poor plant survival leads to lower vegetation coverage which tends to reduce the provision of key ecosystem services from the green roof [7], [10]. Studies of the dynamics of green roof plant species composition show that original species composition shifts over time, with some species becoming extirpated from the system reflecting unsuitability of species to the green roof environment and/or negative interactions with other plant species [11], [12].

Ecosystem services from green roofs depend in part on vegetation composition [13]. Recent work has identified the potential for more diverse plant assemblages to provide enhanced functioning [14], [15], but green roof service provisioning may change as species abundances shift and high species diversity may not persist over time. While there are thousands of plant species that could be used on extensive green roofs, and even more possible combinations of species, horticultural trials to test so many species under controlled conditions would be logistically impossible. To streamline the process of plant selection for green roofs, ecologists have turned to plant traits to determine which general plant characteristics are related to survival, growth and performance of key ecosystem services [16]. Plant leaf traits are commonly used as predictors of ecosystem functioning [17]–[22] and community assembly processes [23], [24]. Leaf and other plant characteristics such as height, flowering phenology, and method of vegetative reproduction have been combined as indicators of general life history strategies, most notably Grime’s Competitive-Stress-tolerant-Ruderal strategies [25], [26]. Such strategies are meant to integrate various components of plant anatomy, morphology, physiology and habitat preferences into general syndromes representing divergent evolutionary responses to the abiotic and biotic environment. In this study, we examined plant species abundance and diversity as it changed over four years in a green roof experiment, and identified leaf traits and life history characteristics that predicted population growth rates (changes in abundance) and final biomass in monocultures and mixed-species plantings. We hypothesized that traits associated with stress-tolerance, such as low stature and low specific leaf area would be the best predictors of growth and biomass in this system.

## Methods

### Ethics Statement

Seeds and leaves were collected from a field site (Duncan’s Cove Nature Reserve) under permit from Nova Scotia Environment; live plants were collected from public but not protected land and no species under protected status was collected. The experiment was carried out on the campus of Saint Mary’s University with permission.

This study examined the changes in species abundance and survival of thirteen perennial plant species in a biodiversity-ecosystem functioning experiment, in a replicated green roof system over four growing seasons (2007–2010) (described previously, see [7]). Each replicate consisted of a black plastic module (36 cm×36 cm×12 cm), with a free-draining base, lined with a composite non-woven water-retention layer (Huesker Inc., Charlotte, NC, USA), followed by an Enkamat (Colbond Inc., Enka, NC, USA) above to act as a drainage/filter layer which was topped with growing medium. We used a commercially available green roof growing medium (Sopraflor X, Soprema Inc., Drummondville, QC, Canada) to a depth of approximately 6 cm (above the Enkamat).

Three species in each of five life-form groups (Table 1) were included in this experiment. Two of the creeping forbs originally included were extirpated by the end of the first year and were not included in any subsequent analyses (these turned out to be annuals that did not re-seed). Total initial density of plants (21) was controlled in each replicate, while composition and/or diversity varied as follows: three replicates of each species in monoculture; five replicates containing each of the three species belonging to a single life-form group; five replicates of each three life-form group combination (ten combinations total), with all three species of each life-form included every time its particular group was included and 20 replicates of the mixture of all five life form groups (15 species initially; 13 species from years 2–4) (Table 1). We planted 21 individual plants in each module (regardless of the number of life-form groups present) in four rows of four plants (on 9 cm centers) and a center row of five plants (on 7 cm centers). For mixtures, we alternated life form groups so that species from a group were well dispersed throughout the module. The planting sequence involved randomly choosing the life-form and species pattern (without replacement) until all species to be included had been selected once, after which the same pattern was repeated throughout the module until a total of 21 plants had been included. Modules were installed on a roof approximately 5 m above the ground on Saint Mary’s University campus in Halifax, Nova Scotia (44**°** 39′ N, 63**°** 35′ W). For more details on roof setting and climate see [7]. Modules were placed randomly into five blocks representing a shading gradient based on proximity of surrounding buildings; block 1 had the least insolation, resulting in the lowest substrate temperatures [27], and average substrate temperatures increase linearly from block 1 to 5. The monocultures only had three replicates each, which were randomly placed into blocks 1, 3, and 5. The complete design consists of 11 levels of the planting treatment factor, which can be grouped into 1-, 3- or 5-life-form treatments, with a random block factor. Response variables (described below) are continuous variables: final above-ground biomass and change in abundance, separately for each species in each replicate.

**Table 1:** A list of all the species used in this experiment including their growth form, the treatments they were used in and their origin.

Species	Code	Growth Form	Treatments	Origin
*Sagina procumbens* L.	Sag. p	Creeping Forb (C)	Mono., C, C/T/S, G/C/S, G/C/T, D/C/S, D/C/T, D/G/S, All	Native
*Danthonia spicata* (L.) Beauv.	Dan. s	Graminoid (G)	Mono., G, G/C/S, G/C/T, G/T/S, D/G/S, D/G/T, D/G/C, All	Native
*Deschampsia flexuosa* (L.) Trin.	Des. f	Graminoid (G)	Mono., G, G/C/S, G/C/T, G/T/S, D/G/S, D/G/T, D/G/C, All	Native
*Poa compressa* L.	Poa. C	Graminoid (G)	Mono., G, G/C/S, G/C/T, G/T/S, D/G/S, D/G/T, D/G/C, All	Introduced
*Empetrum nigrum* L.	Emp. n	Creeping Shrub (D)	Mono., D, D/C/S, D/C/T, D/G/C, D/G/S, D/G/T, D/T/S, All	Native
*Gaultheria procumbens* L.	Gau. p	Creeping Shrub (D)	Mono., D, D/C/S, D/C/T, D/G/C, D/G/S, D/G/T, D/T/S, All	Native
*Vaccinium vitis-idaea* L.	Vac. v	Creeping Shrub (D)	Mono., D, D/C/S, D/C/T, D/G/C, D/G/S, D/G/T, D/T/S, All	Native
*Sedum acre* L.	Sed. a	Succulents (S)	Mono., S, C/T/S, G/C/S, G/T/S, D/C/S, D/G/S, D/T/S, All	Introduced
*Sedum rosea* (L.) Scop.	Sed. r	Succulents (S)	Mono., S, C/T/S, G/C/S, G/T/S, D/C/S, D/G/S, D/T/S, All	Native
*Sedum spurium* M. Beib.	Sed. s	Succulents (S)	Mono., S, C/T/S, G/C/S, G/T/S, D/C/S, D/G/S, D/T/S, All	Introduced
*Campanula rotundifolia* L.	Cam. r	Tall Forbs (T)	Mono., T, G/T/S, D/G/T, D/T/S, G/C/T, D/C/T, C/T/S, All	Native
*Plantago maritima* L.	Pla. m	Tall Forbs (T)	Mono., T, G/T/S, D/G/T, D/T/S, G/C/T, D/C/T, C/T/S, All	Native
*Solidago bicolor* L.	Sol. b	Tall Forbs (T)	Mono., T, G/T/S, D/G/T, D/T/S, G/C/T, D/C/T, C/T/S, All	Native

Mono. refers to the treatment planted with the species in monoculture; Nomenclature follows [Zinck 1998].

Plant communities were maintained by weeding out any species not originally present in the mixture, but new seedlings or vegetative growth from species originally planted were left undisturbed.

Species chosen for this experiment were selected either from naturally occurring habitats in the region (coastal barrens e.g. [28]) or from urban habitats within Halifax, Nova Scotia for the three non-native species: *Poa compressa*, *Sedum acre*, and *Sedum spurium*. These non-natives were included to provide comparisons with species commonly used by industry (the *Sedum* spp.) or with a species commonly occurring spontaneously on European green roofs (*Poa compressa* [29]).

Species abundances were quantified in each year during the biomass peak (late July-early August) using a point intercept method [30], using a pin frame (Domenico Ranalli, Regina, Saskatchewan, Canada). The frame was 30 cm high with a length of 36 cm and a width of 36 cm, contained 16 equally spaced rods (6 mm diameter), and rested on the edge of the module such that the base of the pins was approximately 2 cm above the substrate surface. During sampling we recorded the number of contacts with the rods by parts belonging to each plant species. The sum of contacts was termed the “canopy density” for each species. We calculated the change in abundance (population growth) as (ln(canopy density in year 4) - ln(canopy density in year 1))/# days [31]. All aboveground plant material was clipped at the end of the growing season in year 4, sorted to species, dried and weighed to determine final aboveground biomass for each species in each replicate module. Because several species grew slowly, year 1 canopy density was sometimes zero even though the species was present in the module (it was not large enough to register a contact with the pin frame). To determine change in abundance, these zero values were recoded as 1 contact so that the species could be included in the ln-transformed data. Canopy density for species extirpated from a module by 2010 (not detected in pin frame sampling) was recorded as 0.5 instead of 0, so that we could register a negative change in abundance for such species instead of an undefined value. For calculations of canopy species richness and evenness, these species were left as zeroes. This study addresses only changes in abundance of species, quantified using canopy densities, and final biomass, not other ecosystem functions or services, which are the subject of detailed analyses elsewhere [7].

We determined traits for the 13 species using field sampled material and values from the literature. Five plants per species were randomly selected at a natural coastal barrens site and one leaf was harvested from each plant to determine fresh and dry leaf weights, and leaf area (see Table S1). Non-native plants were sampled from urban populations in Halifax, Nova Scotia. Single-sided leaf area was obtained using ImageJ software (Image Processing and Analysis in Java, http://rsbweb.nih.gov/ij/). Leaves were pressed and dried at 55°C for two days before recording leaf dry weight. Canopy height [26] and plant height were measured in the field. Month of flower initiation, flowering period, maximum height, and lateral spread, following [26] were determined from the literature [32], [33] and corrected based on local field observation if literature values did not correspond with local plant communities. Raw C-S-R (C: competitive; S: stress-tolerant; R: ruderal) scores were derived from the basic leaf and other traits according to [26]. The traits used as predictors in subsequent analyses were leaf area, specific leaf area (SLA: leaf area/leaf dry weight), leaf dry matter content (dry weight/fresh weight), average height and the C, S and R scores. SLA is negatively related to stress-tolerant strategies and positively related to competitive strategies [18], [34]. Stress-tolerant plants are typically short, competitive usually tall while ruderals can vary. Leaf dry matter content is usually negatively associated with growth rates [35].

We compared species richness and evenness (1/Simpson index) separately across all years (combining all planting treatments within a number of life forms (1, 3 or 5)), and for year four species richness and evenness by plotting means and 95% confidence intervals (CI). We used principal components analysis (PCA) to reduce the number of variables and produce composite axes using LA, SLA, LDMC and C, S, and R scores, with variables ln transformed to improve linearity and homogeneity of variance (transformations: ln(LA, SLA, height); square root(LDMC); ln (x+10) used for C,S,R scores). To determine predictors of final biomass and change in abundance in monoculture and the five life-form treatment, we used all-subsets selection using the AICc criterion. Since many models had Δ*_i_* values of below 7 for both response variables, this suggested that there was no best model, so model averaging was used to create a predictive model (using the subset of models with Δ*_i_* values <7)[36], [37]. We separately performed the same analyses using the first three principal components of the trait dataset, but the raw trait values always yielded lower AICc values, so we only used the raw traits in the model fitting. Statistical analyses were completed using the R-package, v. 3.0.2 [38], with model averaging completed using the MuMIn package.

## Results and Discussion

### Changes in Species Richness, Evenness and Abundance

**Figure 1:**
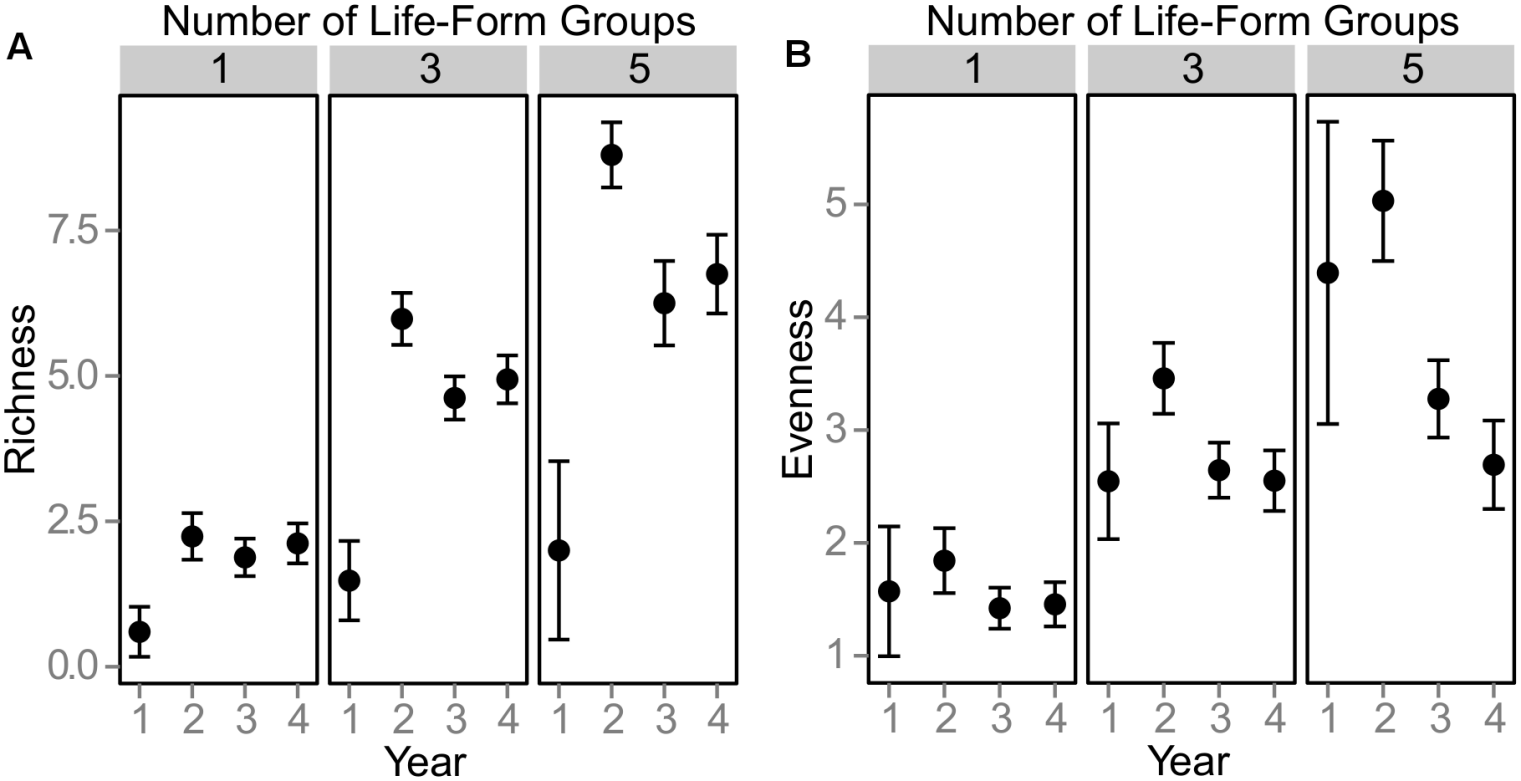
Canopy species richness (A) and evenness (B) over four years (means±95% CI). Treatments grouped by number of life-forms planted; original planted species richness was 3, 9, 15 for 1, 3, and 5 life-form groups respectively. Species evenness is 1/Simpson’s index. Sample sizes: 1 life-form treatements: n = 25; 3 life-form treatments: n = 50; 5 life-form treatment: n = 20.

**Figure 2:**
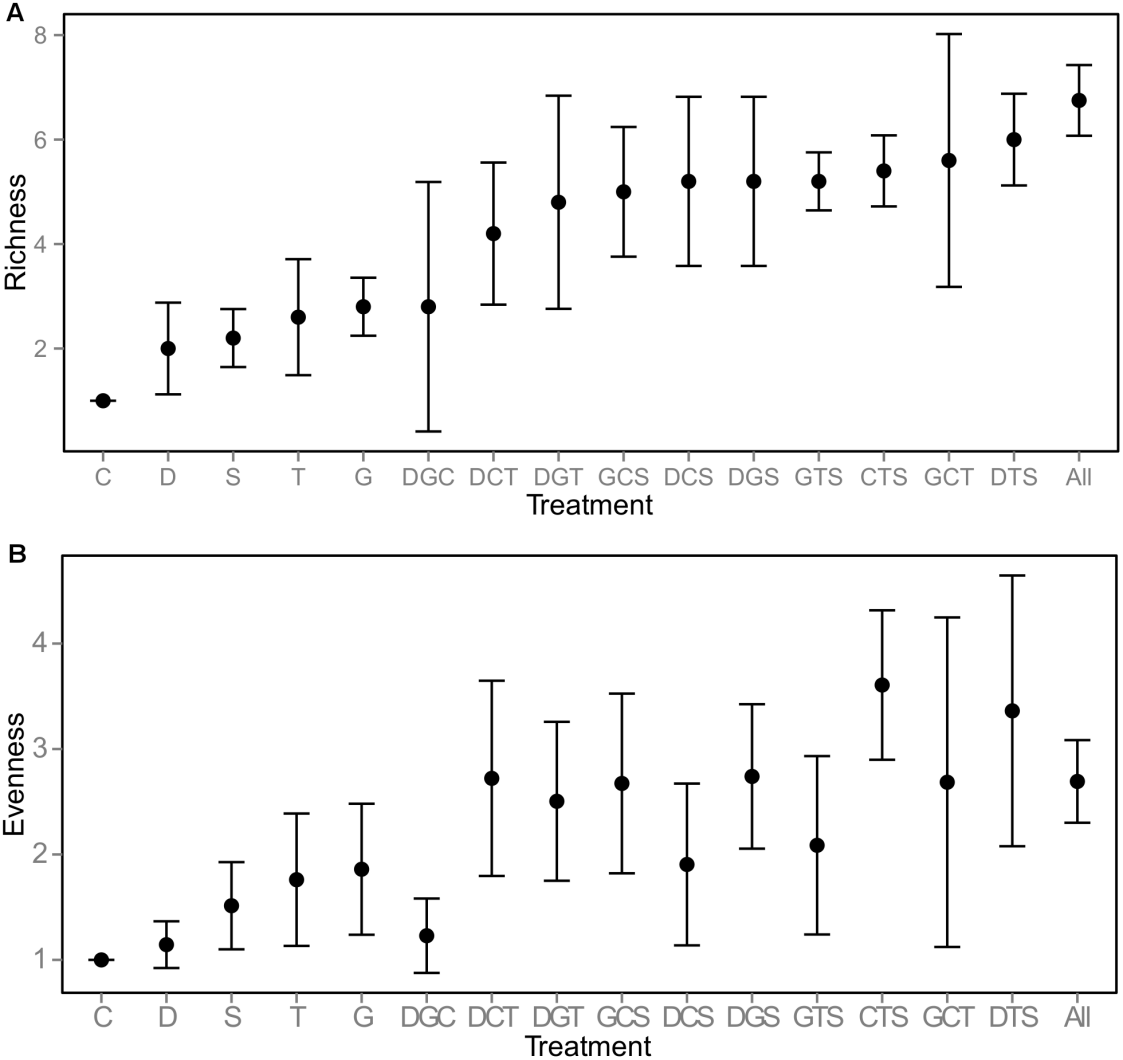
Canopy species richness (A) and evenness (B) in year four, by specific life-form combination (means±95% CI). Sample sizes: 1 life-form treatments: n = 5 each; 3 life-form treatments: n = 5 each; 5 life-form treatment: n = 20.

Canopy species richness and evenness peaked in 2008 (Figure 1). Richness of species detected in vegetation canopies never reached the number of species planted originally in any of the mixture treatments (Figure 1), with the five life-form group (originally planted with 15 species) ending up with an average richness of seven species in the canopy. Single life-form treatments, which started with three species, had equivalent richness (ranging from two to three species) and evenness in year four (Figure 2). The highest richness levels in the three life-form treatments were detected in GCT (grasses, creeping forbs, tall forbs) and DTS (dwarf shrubs, tall forms, succulents) treatments with between five and six species on average. Evenness varied considerably between the three life-form treatments, with CTS (creeping forbs, tall forbs, succulents) having the highest and DGC (dwarf shrubs, creeping forbs, grasses) the lowest. While this method of determining canopy density only samples plants large enough to be detected in the pin frame, the biomass harvest revealed that all species survived in the five life-form treatment (see Table S1) and in most of the other mixture treatments (unpublished data), thus species richness and evenness calculated from canopy density alone are not incorporating all of the species in the community. While canopy biomass and diversity are important predictors of ecosystem functions in this system [7], it is still possible that these smaller individuals with little presence in the canopy and low aboveground biomass could make contributions to overall functioning of the green roof system. At low substrate depths comparable to those in our study, loss of species over time is a common result in green roof studies [11], [12], and competition is frequently cited as the main reason for such declines in richness.

The dwarf shrub treatment ended up dominated by *E. nigrum* by year four, with very little biomass in the canopy contributed from the other two dwarf shrub species (Table S2). All three grass species made up substantial portions of the canopy in the grass single life-form treatment, with *P. compressa* at about double the canopy density as *D. flexuosa*, and 10 x as much as *D. spicata.* In the succulent treatment, final canopy composition was heavily weighted toward *S. acre*, although this species had much reduced canopy density in 2009 (Table S2). *S. rosea* had very low density in the canopy through the entire experiment. The tall forb treatment was dominated by *S. bicolor* throughout, with approximately five and three times the canopy density of *P. maritima* and *C. rotundifolia*, respectively. *S. bicolor* canopy density in the tall forb treatment peaked in 2009. It is possible that weather conditions favoured *S. bicolor* in 2009, while being disadvantageous for *S. acre*. This is borne out in other mixture treatments. *S. acre* was denser in 2008 and 2010 compared with 2009 for the DGS, DTS, GCS, GTS, and five life-form mixture treatments; *S. bicolor* peaked in 2009 in all of the three and five life-form treatments in which it was planted. Weather station data from the site (Table 2) shows that 2009 was substantially wetter than 2008 or 2010. Powdery mildew (Erysiphaceae) was commonly observed on all three succulent species, and it is possible that fungal pathogens associated with wetter conditions decreased growth of *S. acre* in 2009. This finding indicates that fluctuations in species abundance may have been caused by environmental variables, in addition to the successional changes resulting from competition among species. In 2009, when *S. acre* had low abundance in the canopy, the other species in the succulent treatment did not make up for the decline in *S. acre* (see Table S2), but in the treatments with three or five life-forms containing both *S. acre* and *S. bicolor* (CTS, DTS, GTS, CDGST, see Table S2), there was evidence of compensatory dynamics, such that the increase in *S. bicolor* in 2009, the wet year, made up for the decrease in *S. acre*. This suggests another value of planting green roof ecosystems with multiple life-forms: canopy biomass may be more resilient in the face of climatic variation or other disturbances when species or life-forms with contrasting responses to environmental conditions are included.

**Table 2:** Rainfall recorded at green roof site.

Date Range	Total Rainfall* (mm)
June 12-August 31, 2008	298.2
June 12-August 31, 2009	349.9
June 12-August 31, 2010	236.1
April 1-August 31, 2009	639.7
April 1-August 31, 2010	357.6

*Tipping bucket (TE525M, Campbell Scientific, Edmonton, AB) mounted 4 m above roof surface (installed June 2008).

The three life-form mixtures tended to be dominated by *P. compressa*, *D. flexuosa*, *S. acre*, and *S. bicolor* in treatments where these species were planted originally. The treatments including tall forbs with no grasses showed relatively high canopy densities of all three tall forbs (e.g. CTS, Table S2), but when grasses were present, *P. maritima* and *C. rotundifolia* had much lower abundances compared with *S. bicolor* (e.g. GTS, Table S2). *S. procumbens* had low abundances throughout the mixed life-form group treatments. The five life-form treatment was dominated by *P. compressa* by year 4, with over five times the canopy density of the next most abundant species (*D. flexuosa*, *S. acre* and *S. bicolor*). All the other species had low abundances in this treatment, with most showing low value over all four years; *D. spicata* and *P. maritima* had relatively high canopy densities in 2008 but then declined.

**Figure 3:**
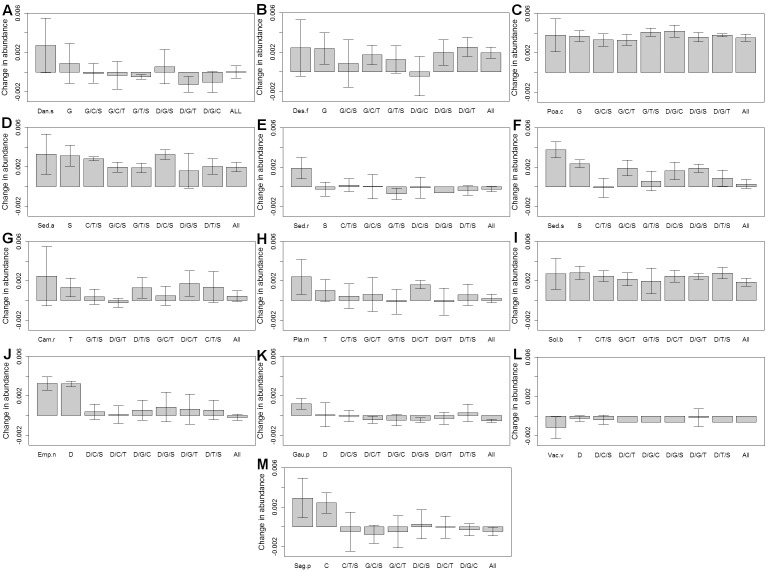
Change in abundance of 13 species in monoculture and mixtures over four years (mean±95% CI). Change in abundance calculated using canopy density: (ln(canopy density in year 4) - ln(canopy density in year 1))/# days. A: *D. spicata*; B: *S. acre*; C: *C. rotundifolia*; D: *E. nigrum*; E: *D. flexuosa*; F: *S. rosea*; G: *P. maritima*; H: *G. procumbens*; I: *S. procumbens*; J: *P. compressa*; K: *S. spurium*; L: *S. bicolor*; M: *V. vitis-idaea*. In each panel, the farthest left bar is the monoculture; mixture life form codes: C = creeping forb; D = dwarf shrub; G = grass; S = succulent; T = tall forb. Sample sizes: 1 life-form treatments: n = 5; 3 life-form treatments: n = 5; 5 life-form treatment: n = 20.

The changes in canopy density in monoculture varied over 20-fold, between *P. compressa* and *S. spurium* canopies growing the fastest, and *V. vitis-idaea*, which had a net negative average change in abundance (Figure 3). Most species had equivalent increases in abundance in monoculture and the corresponding mixture of three species from the same life-form group (Figure 3), suggesting that the net effect of growing with neighbours of the same species is equivalent to that of growing with other species of the same life form group. *S. rosea* was an exception, suggesting a net negative effect of growing with the two other succulents. Most species had a relatively lower change in abundance in the three- and five-group treatments than in monoculture, suggesting a net negative effect of growing with neighbours from other life form groups. In contrast, while *V. vitis-idaea* had negative changes in abundance in all treatments, changes in abundance were less negative in some mixed species treatments compared to the monoculture (Figure 3), suggesting net positive effects of growing with other groups. In this species, individual plants were very small and it seems likely that intraspecific competition was low in the monocultures, and the positive effects of growing with heterospecific neighbours resulted from amelioration of harsh physical conditions (exposure, bare soil) or rather, that lack of plant biomass in the monoculture resulted in conditions that were too exposed, compared with the shelter provided by larger plants in the mixed group treatments. This was the only species that showed a positive response to interspecific neighbours. Given the harsh conditions on extensive green roofs, some authors have suggested that stress-tolerant plants might facilitate other species [39], but only one species out of our 13 showed a positive response to interspecific neighbors.

Five species showed no evident changes in abundance between monoculture and mixture treatments: *P. compressa*, *S. bicolor*, *S. acre, C. rotundifolia, P. maritima*. Of these species, *P. compressa*, *S. bicolor* and *S. acre* had some of the highest positive changes in abundance in monoculture and no apparent net negative effect of growing with other species and groups. *C. rotundifolia* and *P. maritima* showed markedly lower changes in abundance in some mixture treatments, but there was high variability within a treatment; shortages of plant material restricted monoculture treatments to three replicates each. Other species only showed lower changes in abundance in particular group combinations; *D. flexuosa* had low rates of abundance change in the dwarf shrub, graminoid, and creeping shrub mixture but elsewhere exhibited similar changes in abundance to the monoculture. Two tall forbs, *C. rotundifolia* and *P. maritima,* had the lowest abundance growing in a mixture with the dwarf shrubs and graminoids (Figure 3).

### Plant Traits

**Figure 4:**
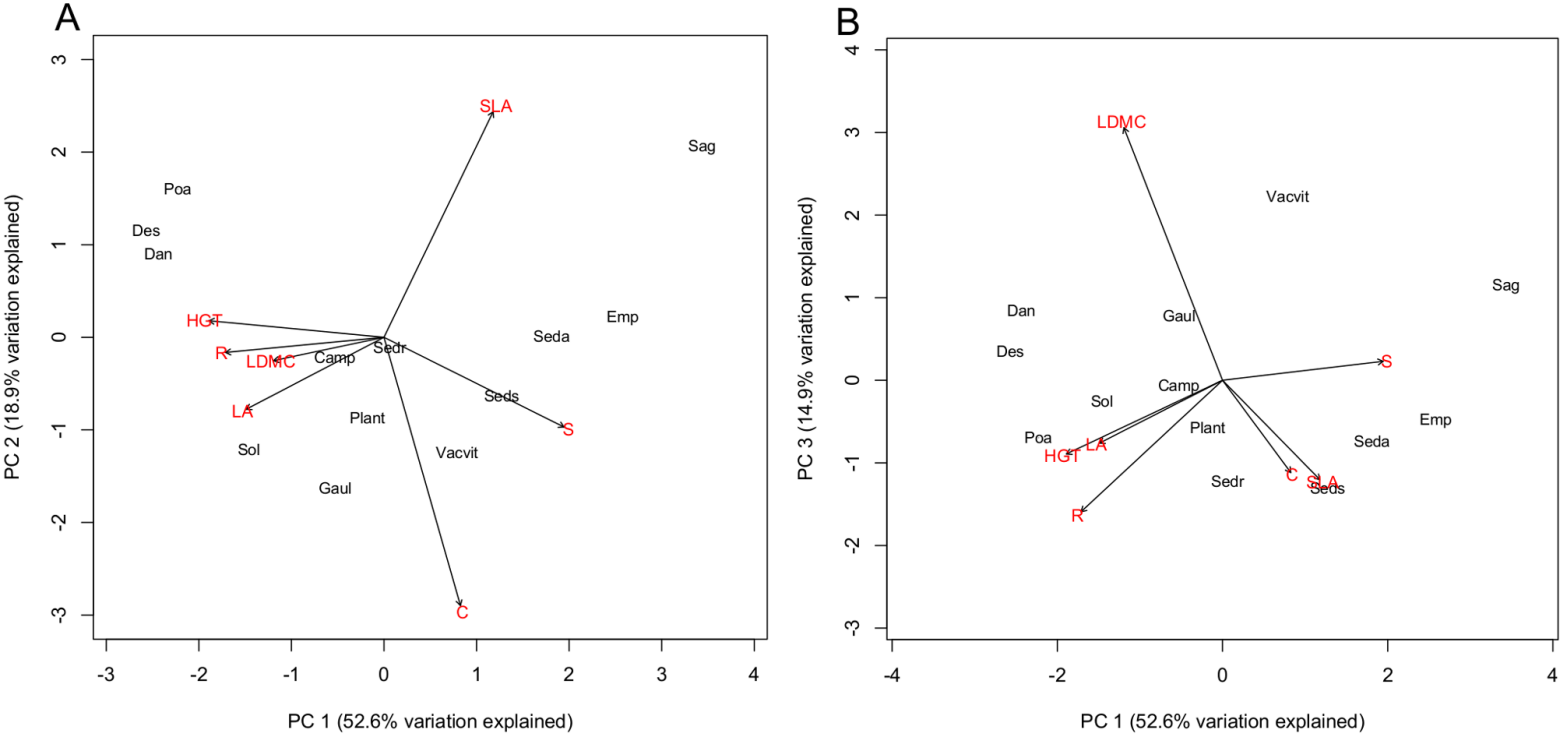
Principal components analysis of species, by leaf traits and life history variables. Species abbreviations: Poa = P. compressa; Des = D. flexuosa; Dan = D. spicata; Traits in red: SLA = specific leaf area; LA = leaf area; LDMC = leaf dry matter content; HGT = plant height; R = ruderal score; S = stress-tolerance score; C = competitive score.

Principal components analysis indicated three meaningful components (eigenvalues >1) (Table S3). The three grass species are very close in trait space, scoring high on the negative end of PC 1, indicating tall plants with relatively large leaf area and ruderal tendencies (Figure 4; Table S1). While these species diverge strongly in their functioning in this system [7], similar heights, leaf traits and other features characterize this group. In contrast, the dwarf shrub group (*E. nigrum*, *V. vitis-idaea* and *G. procumbens*) show greater spread along the first two PC axes, with *E. nigrum* having the highest scores on Axis 1, associated with high stress-tolerance scores, low ruderal scores and low heights. *V. vitis-idaea* and *G. procumbens* have lower axis two scores, indicating lower SLA and greater competitiveness, although these species typically inhabit infertile environments. The other life-form groups show some spread in this trait space. The creeping forb *S. procumbens*, *E. nigrum* and two of the succulents *S. spurium* and *S. acre* have relatively high values of SLA and stress-tolerance. The three grasses have low values of stress-tolerance and high values of SLA. The third principal component is positively correlated with LMDC and negatively correlated with R score. The three grasses show more differentiation along PC 3 with *D. spicata* having the highest LMDC. The dwarf shrub group is also spread out along this axis, with *V. vitis-idaea* loading positively (high LDMC, low C and SLA), followed by *G. procumbens* and *E. nigrum*. Of the species with large positive changes in abundance in both monoculture and mixture *P. compressa* and *D. flexuosa* showed similar aggregate trait values (Figure 3); the grasses, *S. acre* and *S. bicolor* were highly divergent in terms of their trait values, suggesting that high positive rates of change in abundance in this system can be attained by different trait combinations. The native species in this category are typically found in the periphery of coastal barrens heathland habitat where disturbances open up the matrix of dominant ericaceous plants, whereas *P. compressa* and *S. acre* are commonly found in disturbed, hard-surfaced environments in urban areas. The dwarf shrubs, dominant on organic soils on coastal barrens [28], showed lower changes in abundance when combined with the other species suggesting lower competitive ability in the green roof system. The growing medium used in this experiment was designed for extensive green roofs planted with *Sedum* spp., and had a relatively high pH, low organic matter content and high soluble nutrient concentrations, in contrast to the native soils where the dwarf shrubs dominate [7], [28]. With a more suitable substrate, we might expect these native shrubs to grow faster, and perhaps to out-compete ruderal species that are adapted to more fertile conditions. Nevertheless, high changes in abundance in *S. bicolor* and *D. flexuosa* suggest that native species can have high positive changes in canopy abundance in a green roof system, if suited to substrate properties.

SLA was a strong positive predictor of final biomass in monoculture (Table 3); R and S scores were positive and negative predictors, respectively, in both monoculture and the five life-form treatments. In general, the traits predicted biomass in both monoculture and five life-form treatments in the same directions but with variation in the size of the coefficients (Table 3). Coefficients for LA, LDMC and C scores in the biomass models were weakly negative or close to zero, those for height were positive.

**Table 3:** Multiple linear regression showing standardized coefficients from model averaging for final biomass and change in abundance in monoculture and five life-form treatments.

Dependent Variable	Predictors	Model Averagedβ Coefficient	95% ConfidenceInterval Lower bound	95% ConfidenceInterval Upper bound
**Monoculture** **biomass**	**SLA**	0.73	0.30	1.16
	**LA**	−0.11	−0.57	0.35
	**LDMC**	0.09	−0.36	0.54
	**Height**	0.41	−0.22	1.03
	**R score**	0.63	0.16	1.04
	**S score**	−0.43	−0.94	0.07
	**C score**	−0.19	−0.68	0.30
**Five life-form** **treatment biomass**	**SLA**	0.39	−0.03	0.81
	**LA**	0.02	−0.49	0.53
	**LDMC**	−0.25	−0.76	0.25
	**Height**	0.24	−0.54	1.03
	**R score**	0.68	0.18	1.18
	**S score**	−0.61	−1.15	−0.07
	**C score**	0.04	−0.49	0.58
**Monoculture change** **in abundance**	**SLA**	0.76	0.29	1.23
	**LA**	0.10	−0.43	0.64
	**LDMC**	−0.60	−1.10	−0.09
	**Height**	0.53	−0.07	1.14
	**R score**	0.53	0.11	0.94
	**S score**	−0.61	−1.14	−0.07
	**C score**	0.21	−0.31	0.73
**Five life-form** **treatment** **change in** **abundance**	**SLA**	0.48	0.02	0.94
	**LA**	0.37	−0.19	0.94
	**LDMC**	−0.14	−0.75	0.48
	**Height**	0.30	−0.57	1.18
	**R score**	0.63	0.09	1.16
	**S score**	−0.62	−1.18	−0.06
	**C score**	−0.07	−0.69	0.55

The models for change in abundance had relatively high positive coefficients for SLA and R score in both monoculture and the five life-form mixture (Table 3). LDMC and S score were strong negative predictors of change in abundance in monoculture, and height was a relatively strong positive predictor. C score and LA were weaker positive predictors. Change in canopy abundance in the five life-form treatment had positive coefficients for LA and height, weak negative coefficients for LDMC and C score, and a strong negative S score.

While we expected more stress-tolerant species to perform the best in this shallow-substrate environment, we suspect that the high nutrient levels and pH of the green roof substrate gave the more ruderal species an advantage, leading to shifts in species composition over time. While this was not quantified, many of the species that we weeded out of the modules over the experiment were non-native, ruderal species common in urban areas, further supporting the idea that this particular green roof system in this climate promotes the growth of ruderals over stress-tolerant species or “faster” over “slower” species (*sensu* [40]), at least in the first four years post-establishment. It should also be pointed out that we only examined aggregate changes in abundance over the entire four years. Early establishment growth during year 1 may reflect a different environment, dominated by abiotic factors such as high soil temperatures and moisture limitations, whereas growth in later years may reflect plant responses to more intense competition with intra- or interspecific neighbours. These possible effects cannot be sorted out with these data as initial post-planting sampling resulted in many zeroes in the canopy density measurements, so initial vs. end of year 1 comparisons would result in unreliable change in abundance calculations, and were not included here. Longer-term studies of green roofs could be expected to show greater dominance of stress-tolerant species over time, as nutrients become more limiting, but empirical studies in Europe show that a suite of ruderals dominates after 20–100 years [29], [41], including some dominant in our study (*S. acre* and *P. compressa*). While the regressions suggest that species with ruderal traits have the highest increases in abundance here, *S. acre* was associated with higher S scores, but also had high change in abundance. Overall, SLA predicted changes in canopy abundance and biomass in monocultures and mixtures and could be used to select species for extensive green roofs in this climate, in fertile substrates. Reich [40] posits an overall differentiation of plant species along an access of “slow” to “fast”, referring to the rates at which they can acquire resources, with slow plants dominating in low resource areas, which have been characterized by others as stressful [25], and faster plants dominant in more resource-rich environments. The traits that predicted successful growth and biomass accumulation were similar in the monocultures and the mixture combining all the species, and were more strongly related to “fast” traits than those possessed by species typically growing in low-resource environments. Increases in abundance in the mixtures occurred primarily between years 1 and 2 for most species.

All mixture treatments ended up with fewer species in the canopy than originally planted, and the change in abundance results indicate that the majority of species performed poorly in multi-species mixtures. While environmental fluctuations can account for some of the year-to-year variation, we suggest that most of these species experienced net negative effects of interspecific competition. The species that performed well in both monocultures and mixtures were predicted by leaf and aggregate life history traits, suggesting that this method can be used to select green roof plant species. However, if high diversity in green roof systems is to be maintained, selection of species that can coexist, alterations to substrate conditions to favor more stress-tolerant natives, or incorporation of spatial heterogeneity into the system might all help promote longer-term species coexistence.

## Supporting Information

Table S1 **Plant trait values for each species.**
(XLSX)

Table S2 **Canopy density for each species in each treatment over four years.**
(XLSX)

Table S3 **Details of principal component analysis of plant traits.**
(DOCX)
