# Supplementary material for: Leaf and Life History Traits Predict Plant Growth in a Green Roof Ecosystem
Source: PLoS One. 2014 Jun 30;9(6):e101395. doi: 10.1371/journal.pone.0101395 (PMC4076323; doi:10.1371/journal.pone.0101395)
Supplement: Table S3 — Details of principal component analysis of plant traits. (DOCX) [file pone.0101395.s003.docx]

Table S3. Principal components analysis of plant traits.

|  | PC1 | PC2 | PC3 | PC4 | PC5 | PC6 | PC7 |
| --- | --- | --- | --- | --- | --- | --- | --- |
| Eigenvalue | 3.68 | 1.32 | 1.04 | 0.54 | 0.22 | 0.16 | 0.03 |
| Proportion  variance explained | 0.53 | 0.19 | 0.15 | 0.08 | 0.03 | 0.02 | 0.01 |
| Original variable | Loadings on Principal Components | | | | | | |
| S | 0.49 | -0.24 | 0.06 | -0.05 | 0.17 | 0.41 | 0.71 |
| C | 0.21 | -0.72 | -0.28 | -0.04 | -0.43 | 0.41 | -0.03 |
| R | -0.43 | -0.04 | -0.40 | -0.33 | -0.42 | 0.61 | 0.04 |
| Height | -0.47 | 0.04 | -0.22 | -0.33 | 0.29 | 0.49 | 0.54 |
| Leaf area | -0.37 | -0.19 | -0.19 | 0.86 | 0.07 | -0.11 | 0.19 |
| Specific leaf area | 0.29 | 0.61 | -0.30 | 0.21 | -0.52 | 0.22 | 0.29 |
| Leaf dry matter content | -0.29 | -0.06 | 0.76 | 0.02 | -0.49 | 0.07 | 0.27 |
